# A Qualitative Descriptive Analysis of Spiritual Care Practices Among Advanced Practice Nurses

**DOI:** 10.1155/jonm/8169809

**Published:** 2026-11-05

**Authors:** Abigail Kra-Friedman, Nasreen Lalani, Gulnar Ali, Caryn Scheinberg-Andrews, Joanne Pike, Kimberley Lamarche, Elisabeth Gulliksen, Melanie Rogers

**Affiliations:** ^1^ Department of Nursing Ashkelon Academic College Ashkelon Israel ash-college.ac.il; ^2^ Selma Jelinek School of Nursing Jerusalem College of Technology Jerusalem Israel jct.ac.il; ^3^ School of Nursing Purdue University West Lafayette Indiana USA purdue.edu; ^4^ Department for Social Work Counselling and Social Care University of East London London UK uel.ac.uk; ^5^ Hadassah Henrietta Szold School of Nursing Hebrew University of Jerusalem Jerusalem Israel huji.ac.il; ^6^ School of Nursing Wrexham University Wrexham UK; ^7^ Canadian Nurses Association Ottawa Canada cna-aiic.ca; ^8^ Department of Nursing University of Huddersfield Huddersfield UK hud.ac.uk

**Keywords:** advanced practice nurse, international, qualitative, spirituality

## Abstract

**Aim:**

This study examined how international Advanced Practice Nurses (APNs) understand and incorporate spiritually competent practice across diverse clinical environments, alongside Rogers’ *Availability and Vulnerability Framework*.

**Background:**

Spirituality plays a vital role in the delivery of holistic care by APNs, yet it remains a concept that is often difficult to define and is frequently neglected in clinical settings.

**Methods:**

As part of a larger mixed‐methods study, 13 APNs from around the world took part in semistructured interviews conducted in 2023. Participants were asked about their perceptions of *spiritually competent practice* and the *Availability and Vulnerability Framework*.

**Results:**

A qualitative descriptive concept‐driven deductive approach revealed four primary themes: f*oundation of universal spiritual principles, implementation of universal spiritual principles in clinical practice, challenges in the operationalisation of spiritual care* and m*ental health capabilities enabling spiritual care*. The concepts found in the definition of *spiritually competent practice* and Rogers’ *Availability and Vulnerability Framework* were found to be central to exploring how spirituality might be more effectively integrated into APN practice.

**Conclusion:**

Findings revealed ongoing obstacles to integrating spiritual care, underscoring the need for its explicit, deliberate implementation at the system, management and leadership levels, as well as its inclusion in APN educational curricula.

**Implications for Nursing Management:**

This study provides valuable insights for nursing management by demonstrating the impact of spiritually competent practice on patient care, provider well‐being and operational efficiency, thereby informing evidence‐based organisational standardisation and management practices. Policy makers should recognise the value of spiritual care and ensure adequate staffing, funding, support, continuing education and time to enhance integration into routine practice. Ultimately, the study highlights the importance and value added of preparing APNs with the insight and tools necessary to provide spiritual care that genuinely addresses the health of the whole person.

## 1. Introduction

Advanced Practice Nurses (APNs) work within an expanded scope of practice to provide direct patient care, improving the health and well‐being of individuals, families and communities across all clinical settings [1]. This practice is holistic, bringing together the bio‐psycho‐social‐spiritual approach to care. The EPICC network defines spirituality as ‘the dynamic dimension of human life that relates to the way persons (individual and community) experience, express and/or seek meaning, purpose and transcendence and the way they connect to the moment, to self, to others, to nature, to the significant and/or the sacred’ [2]. Whilst this definition is clear, what is less clear is what we mean by the provision of spiritual care with the limited literature on what this means for APNs. Whilst the literature does acknowledge the need to include spirituality in holistic practice and APN competencies [3–5], there are gaps and barriers in understanding if APNs integrate spirituality and spiritual care into care settings.

A growing body of evidence underscores the impact of spiritual care interventions on patient outcomes. In an umbrella review of 27 studies exploring 14 interventions, researchers found that providing spiritual care to patients increases their quality of life improves emotional and physical symptoms and overall spiritual well‐being [6]. In another meta‐analysis of 28 randomised studies, researchers found similar results [7]. Lastly, in a seminal systematic review on spiritual care and mental health, spiritual care was shown to reduce depression, anxiety and psychological distress, while acting as a protective factor against suicide and substance use disorders [8]. Most existing studies have focused on nurses rather than APNs, leaving a notable gap in the understanding of spiritual care within advanced practice nursing. Moreover, no studies were found that asked APNs about their perceptions of models or frameworks for implementing spiritual care which would inform their practice.

To address this gap in the literature, this study explored international APNs’ perceptions of their own spirituality, practices for spiritual care and their perceptions of Rogers’ [5] *Availability and Vulnerability (A&V)* Framework and Wattis et al.’s [9] definition of *Spiritually Competent Practice (SCP).* A secondary aim was to make recommendations for how nurse managers may advocate for spiritual care in APN practice settings.

### 1.1. Availability and Vulnerability Framework

Figure 1 reflects Rogers’ [5] *Availability and Vulnerability (A&V)* Framework. The framework offers a comprehensive approach to understanding human‐to‐human interactions within the context of APN care. Availability refers to the APN’s openness to being present with the patient in an authentic and empathetic manner. Vulnerability involves acknowledging one’s limitations, which helps build trust and foster a supportive environment where the patient feels valued and understood. Through these principles, APNs create a therapeutic relationship that encourages emotional connection and healing among patients, families and providers.

**FIGURE 1:**
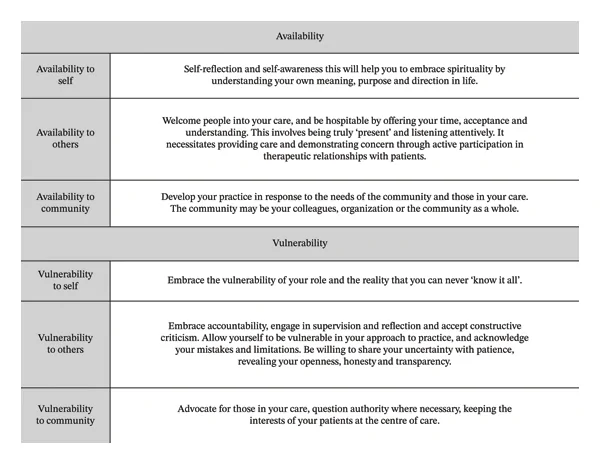
Rogers’ [5] availability and vulnerability framework.

The framework aims to extend beyond the individual APN‐patient relationship to encompass broader social and cultural contexts. It recognises that human experiences are interconnected and influenced by different levels of interaction. At the *self-level,* the framework emphasises self‐awareness and reflection, while at the level of the *other*, it stresses the importance of interpersonal relationships. At the *community* level, it acknowledges the societal and cultural factors that shape care. By applying the A&V Framework, APNs can enhance their practice, build stronger patient connections and provide more compassionate and effective care.

### 1.2. Spiritually Competent Practice (SCP)

SCP, as seen in Figure 2, is described as the ability of healthcare practitioners to recognise, respect and address the spiritual needs of their patients as an integral part of holistic care [9]. This involves more than just acknowledging a patient’s spiritual beliefs; it requires actively engaging with, and incorporating them, into healthcare planning and delivery. Practitioners must be aware of their own spiritual values and potential biases, ensuring that patient care is respectful, individualised and free from judgement.

**FIGURE 2:**
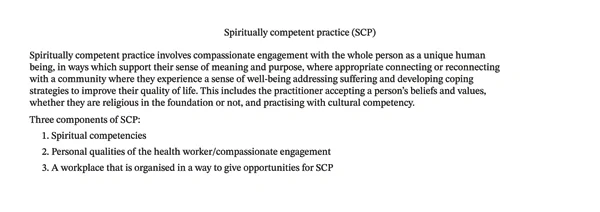
Definition of spiritually competent practice ([9], pp. 53–69).

According to Wattis et al. [9], SCP is a dynamic and ongoing process that necessitates continuous learning and reflection. Healthcare practitioners need to adapt to their patients’ diverse spiritual needs, which can improve patient satisfaction, foster trust and contribute to a more comprehensive approach to healing. They emphasise that spiritual competence requires knowledge and training. The EPICC Network Spiritual Care Education Standard and resources on their website can support knowledge development [2]. A deep commitment to treating spirituality as a fundamental aspect of holistic care underpins the personal qualities of practitioners. Whilst the workplace must recognise the importance of spirituality in healthcare and support practitioners to integrate this into their practice. SCP guided by the A&V framework can address and promote the integration and practice of spiritual care for APNs, resulting in a meaningful human‐centred care approach.

## 2. Methods

### 2.1. Design

This qualitative descriptive study was part of a larger mixed‐methods exploration of APN spirituality. Qualitative descriptive studies aim to identify, analyse and interpret patterns of meaning or themes within the data [10]. The focus is on providing a rich, comprehensive summary of a phenomenon rather than explaining it. This approach, often used in nursing research, is designed to capture the who, what, where, and how of a research topic in a straightforward, journalistic manner. Unlike phenomenological, ethnographic, or grounded theory studies that aim to interpret or develop theories, qualitative descriptive studies seek to describe experiences directly, making this approach useful for exploring poorly understood phenomena [10].

Studies that utilise this approach are often flexible in the data collection and analysis phases [10]. Semistructured interviews are commonly used because they offer a reliable method for gathering rich, descriptive data. Analysis tends to be more *categorical* and less *conceptual*, often utilising qualitative content analysis to organise findings. The strength of this approach lies in its ability to present clear, practical insights.

Ethical approval was granted by the University of Huddersfield (SREIC/2021/105) and the University of East London, UK (Co‐I) (2023).

### 2.2. Characteristics of the Research Team

The research team included six senior female PhD‐prepared nursing researchers from four different countries with extensive experience in qualitative research, prior study of spiritual care and cultural awareness training. Six members of the team were APNs in various specialities, including women’s health, adult primary care and oncology. Two PhD students and two BSN students worked on data collection and analysis with one PhD researcher (J.P.). Team members conducted interviews in English to ensure standardisation and consistency using a shared semistructured interview guide. The research team met regularly to ensure standardisation. Final analysis was completed by PhD researchers A.K‐F., C.S‐A. and M.R.

### 2.3. Reflexivity

All authors are APNs who provide and research spiritual care, positioning the team with substantial domain expertise but also introducing potential for shared assumptions and interpretive bias. One author (M.R.) developed the A&V framework used in this study, which participants reviewed in advance, thereby increasing the risk of confirmatory bias. The team therefore engaged in ongoing reflexive practice, explicitly acknowledging how their professional positioning and investment in the framework could shape data collection and interpretation.

To mitigate bias, the researchers maintained an audit trail documenting analytic decisions and held regular meetings to critically discuss coding, challenge assumptions and consider alternative interpretations, including disconfirming data. These strategies supported transparency and helped ensure that findings were grounded in participants’ accounts rather than driven by pre‐existing frameworks.

### 2.4. Participants

A convenience sample was recruited from volunteers who completed the quantitative survey in the parent study and volunteered to participate in the qualitative interviews. The parent study employed convenience snowball sampling through professional APN networks, organisations, associations and personal contacts. All participants of the parent study were invited to participate. To meet inclusion criteria, participants had to meet the definition of an APN as defined by the International Council of Nurses [1], be fluent in reading/writing English, and have access to a computer with an Internet connection. The sample included nurse practitioners and clinical nurse specialists representing a range of clinical specialities and WHO world regions.

### 2.5. Procedure

Quantitative survey data were collected over a 4‐month period in 2022 and reported elsewhere (Rogers et al. 2025) and included an item inviting respondents to participate in follow‐up qualitative interviews. Semistructured individual interviews were conducted by research assistants who had 1–1 supervision and training; interviews were conducted online via the Microsoft Teams platform between July and September 2023. Data were securely stored at the University of Huddersfield and will be retained for 10 years in accordance with institutional data governance requirements.

Interviews lasted approximately 40–60 min, were audio‐recorded with consent and transcribed verbatim. All data were anonymised, and alphabetised pseudonyms were assigned to participants. Prior to the interview, following consent, participants received Rogers’ [5] A&V Framework (Figure 1) and Wattis et al.’s [9] definition of SCP (Figure 2) via email to prepare for the interview. Demographic characteristics and APN background information were collected at the beginning of each interview.

A semistructured interview guide was used to explore participants’ perceptions of meaningful aspects of practice, understandings and expressions of spirituality, and approaches to integrating spirituality within their clinical roles. Participants were additionally invited to reflect on the SCP definition and the A&V framework and to comment on their applicability and usefulness in clinical practice.

### 2.6. Data Analysis

This study employed the steps as outlined by Villiman et al. [10] using a concept‐driven deductive approach to expand or test Rogers’ [5] *Availability and Vulnerability Framework* and Wattis et al.’s [9] definition of *spiritually competent practice* against the collected data. Meaningful codes were extracted from the transcribed interview data and organised into subthemes and then further developed into core themes, ensuring the findings captured both individual experiences and broader shared contexts. Data collection and analysis were completed when thematic saturation was reached and no new concepts were found.

An iterative analysis for expanded perspective was performed by J.P., C.S‐A. and A.K‐F. A reflexive process facilitated further critical questioning and reflection on identifying and naming the themes, which were then revisited and confirmed by the rest of the research team, ultimately leading to the consensus on the findings described below. This collaborative approach ensured that the findings were meaningful to the participants and understandable to the broader audience.

## 3. Results

### 3.1. Characteristics of the Participants

The study included a diverse group of APNs from multiple international settings. Participants were predominantly female (*n* = 12), with one male participant, and ranged in age from 39 to 74 years. They represented a wide geographic distribution, including the United Kingdom, United States, Canada, Australia, New Zealand, Malta, China, Kenya, Ghana and India (Bangalore). Areas of practice were varied and included mental health, family practice, paediatrics, nutrition, pain services and advanced surgical and anaesthesia roles, with several participants also working as educators and researchers. Years of APN experience ranged from 4 to over 40 years, reflecting a highly experienced cohort overall. Participants were assigned pseudonyms for anonymity.

### 3.2. Findings

Findings were analysed manually and reported using the Consolidated Criteria for Reporting Qualitative Research (COREQ) checklist [11]. Digital audio recordings of 13 interviews lasting between 40 and 60 min were transcribed. Participants were asked to describe their own spiritual practices and how they incorporate their spirituality into their clinical practice and to reflect on the SCP definition and A&V framework. Interestingly, participants all described living their lives by universal spiritual principles. Additionally, they agreed that the SCP definition and the A&V framework were clear and helpful. Almost all participants noted the challenges in implementing and operationalising the definition and framework using measurable outcomes and the difficulty in teaching the concepts to APN students. Participants also noted the conflation of spiritual principles with essential interpersonal and emotional skills commonly used in healthcare.

Table 1 shows the findings of the analysis, which resulted in 71 codes that were further collapsed into ten subthemes and four themes. The four themes included f*oundation of universal spiritual principles, implementation of universal spiritual principles in clinical practice, challenges in the operationalisation of spiritual care* and *mental health capabilities enabling spiritual care*.

**TABLE 1:** Findings.

Codes	Subtheme	Theme
Vulnerability not weakness	Intrapersonal attributes	Theme 1: foundation of universal spiritual principles
Openness to change—pivoting		
Authentic and vulnerable—are they the same thing?		
Reciprocation		
Admitting we are not experts		
Admitting we are not in control of everything		
Share successes with patients and other team members		
Work towards balance		
Humility		
Emotional intelligence		
Being hopeful		
Transcendence		
Deeper sense of self	Role of spirituality in the practitioner’s life	
Constant spiritual growth		
Evolving over time		
Spirituality permeates everything I do		
Spirituality is subtle and humble		
Underlying spiritual principles		
If my spirituality erodes, I become less effective		
Flourishing life		
Letting go of control		
Spirituality is like a muscle		
Usefulness to others		

Creating meaningful care	Spirituality as connection	Theme 2: implementation of universal spiritual principles in clinical practice
Making connections		
Connection with a higher power		
Really seeing the person		
Connection at every level		
Appeal to the spirit		
Sharing my weakness with others		
Active listening	Interpersonal practices	
Cultural humility		
Respectful care		
Compassionate care		
Value‐based care		
Accepting		
Dignified care		
Flexibility		
No judgement		
Asking others: What do you think?		
Modesty		
Being teachable		
Admitting we are not in control (people, disease processes, outcomes, successes)		
Meeting people where they are		
Receiving and giving		
Creating a sense of ease and comfort		
Advocacy		

Needs to be explained and operationalised	Operationalisation	Theme 3: challenges in the operationalisation of spiritual care
Framework and definition are superficial because they are hard to operationalise		
Lack of clarity in frameworks		
Complicated	Integration of spiritual care	
Hard to implement		
Need concrete instructions/exercises/curricula		
No assessment tools—not a checklist		
Takes too much time to do it		
APN students not learning about spirituality	Learning and teaching spiritual care	
Introduce SCP into the basic curriculum		
Need to teach APNs spirituality for themselves and their patients		
No one taught me this		
No mentorship available		
How do you teach connection?		
No difference between religions	Bureaucratic, management, legal, structural, cultural and religious challenges	
Religion as a tool to connect		
Consistent with Jesuit values		
Maori tradition		
Respect separation of church and state		
Use of religious terminology in practice		
How do I explain this to my supervisor?		
My organisation does not support spiritual care		
Regulatory barriers		
Need to use code words because cannot say anything about religion		

Spiritual care requires the same skillset as mental healthcare	Similarity to mental health providers’ skillset	Theme 4: mental health capabilities enabling spiritual care
Psychosocial skills		
Building therapeutic relationships		
Mutuality in care		
Buoying up patient’s spirits		
Hidden in mental health	Clinician well‐being and resilience	
Caring for the carer		
Preserving mental health		

### 3.3. Theme 1: Foundation of Universal Spiritual Principles

This theme refers to the many universal spiritual principles that underlie the central role spirituality plays *in participants’ own lives*. Codes included examples of *intrapersonal attributes* (as seen in Table 1) such as authenticity, humility, reciprocation, relinquishing control, transcendence and finding hope, which gave participants a sense of balance and inner calm. APNs noted that these attributes and principles were not unique; they may be found in many religions, cultures and spiritual frameworks from around the world.

**Laura** from the United States of America suggested: ‘[Spirituality is] *included in multiple religions…there are many pathways toward a spiritual life and many wisdom traditions…the religious contemplative…moral ……environmental… [which] develops your own truest self and is in accordance with these fundamental virtues and…whatever one’s understanding of a greater power….energetic field …love force …source of being’.*

While **Charlie** from Canada felt that: ‘*At the heart of all religions is there’s a spiritual truth. It’s ultimately the source of my wellbeing. It’s at the heart of pretty much everything in life…really permeates everything that I do …Just kind of respecting the mystery of our own existence and trying to connect with something that feels like a higher intelligence. Seeing that connection that’s already there…the deeper spirituality is the sense of loving people…. The idea of servicing the higher good. … much more than a belief system’*.

Others identified aspects of transcendence:

“It’s broader, it’s more about my relationship to the universe, that has pieces of the former religion and then other religions as well” **(Helen** from Canada**).**

*“I consider spirituality something which is very high and beyond religion.”* **(Ben** from Malta**)**

Spirituality was not seen as a peripheral aspect, rather as a central aspect of human identity; participants noted that spirituality was deeply embedded in their personal and professional lives. For many APNs spirituality seemed to be a vital force that permeated everything they do in an ongoing process of growth and self‐discovery. This spiritual journey is not static but continually evolving, profoundly shaping their inner selves, echoed by **Emma** from Australia: ‘*Spirituality is a sense of being. There is a being greater than we are, inside of us…. this greater wisdom. It’s like the light that ignites us…It’s honouring everything and where we are. Being grateful. In the trees, in the rocks, in the flowers, in the rain. It gives us rules, the good and the bad’.*

Spirituality was also understood as something that requires nurturing and constant engagement, like a muscle in the body, which APNs must consistently develop to maintain their ability to connect with a higher power, themselves and others. **Charlie** recognised that: ‘*If the spirituality piece erodes too much, then I become less effective in every aspect of my lif′e’.* Within this theme, vulnerability, openness and humility further emphasise the importance of APNs maintaining a humble, open approach. Being vulnerable is seen not as a weakness but as an opportunity for authentic patient engagement.

Participants described spirituality as a deeply personal relationship and connection between oneself, surrounding individuals and a higher power, conceptualised as God for some. This relationship is characterised by safety, protection and a sense of belonging. Spirituality can encompass diverse belief systems and practices, reflecting individual experiences and perspectives. Participants emphasised the importance of authenticity in spiritual exploration, acknowledging the uniqueness of each person’s spiritual journey as integral to holistic well‐being, encompassing the physical, emotional and spiritual dimensions of health. **Fran** from Australia recognised this: ‘I *think it’s important to be a good person and treat people the way that I, my relatives, friends would want to be treated… forgiveness, cultivating the ability to understand …’.* The APNs highlighted their competency in honouring oneself, others and nature in spiritual practice, fostering a sense of respect, empathy and interconnectedness. **Anabel** from India stated: ‘*Spirituality is the… relationship between… myself… surrounding people and the supreme power… God. If I can say spirituality is a state of full mind which will help us to be in relationship with our surroundings and our creator’*. Intrapersonal competencies in these areas constitute SCP. Participants showed awareness of the meaning of spirituality to themselves and demonstrated a strong sense of connectedness to self and others.

### 3.4. Theme 2: Implementation of Universal Spiritual Principles in Clinical Practice

This theme reflects going beyond simple interventions to the heart of holistic APN practice and creating *deep and meaningful interpersonal connections* with patients. They describe the act of seeing the whole person, beyond the clinical diagnosis, offering care that reaches the spiritual, emotional and physical dimensions. **Ben** reflected on this when considering his interactions with elderly patients: ‘*Elderly people clutch on to spirituality. It’s sort of their saviour, you know? And it’s important to include it in their care when you’re talking to them … the way you act with them, you know… you give them time to pray… you give them time to be on their own and be calm and… So, it’s very, very important… they need a niche where they can feel safe. It involves even including the family’.*

Participants indicated that the interpersonal interaction between the APN and patient includes a deep connection with a higher power. **Anabel** revealed this: ‘*I believe that the effect of communication skills through talking and listening means effective communication are the key cornerstone to the therapeutic relationship…spirituality is a state of mind that will lead us to be in harmony, in harmony with the surrounding people and with our creator, and with our God’.*

Through implementing spiritual care, APNs reflected on their own vulnerability and identified that they share their personal experiences with patients when appropriate, which they felt enriched the relational dynamic and fostered a more profound, holistic approach to care.

This theme also describes how the APNs used *interpersonal spiritual attributes* in direct patient care. These included: active listening, respect, compassion and meeting patients where they are, allowing for a personalised approach to care. These foundational approaches highlight a deep commitment to value‐based, dignified care, where the APNs recognised the importance of flexibility, nonjudgement and a willingness to learn from patients. **Emma** embodied this: ‘*I come to people, really accepting them where they are, not trying to change them I’m basically here to consult in the background. ‘A lot of people don’t accept themselves, but my accepting them gives them comfort’*. The APN’s role is not to impose their own beliefs but to create an environment of acceptance where the patient’s spiritual needs can be explored and honoured. **Debbie** from China recognised this: ‘*Communication with patients* (is essential), *to enhance the trust of patients, to maintain a good attitude and listen to the inner thoughts of the patients and the emotional change of the patients. There is a sense of well-being addressing suffering to improve their quality of life and accepting their values. Creating a safe place. Being compassionate’.* **Kay** from Ghana described ‘*many opportunities to bring [spirituality] in…sometimes people want to have a religious item. And if it’s safe to do that in the surgery…sometimes I’ll let them’*.

Participants’ approach to care incorporated seeking to understand the whole person, not just their physical symptoms, but also their spiritual and emotional well‐being. **Charlie** described dealing with difficult patient visits: ‘*My purpose is just servicing the higher good and not seeing the other person as this difficult person, but just somebody that needs assistance…* [this] *keeps you fresh, keeps you inspired, … before I call, because I know it’s going to be a hard encounter… I’ll pray for a few minutes just to get into the right headspace before I enter … Sometimes even during lunchtime, I’ll sit down to meditate and just regroup myself to try to have a good afternoon and be as effective as possible for the clients’.*

**Emma** also took time to ground herself: ‘*Between each patient, I consciously take a deep breath, and I put my hands on my chest. When I feel these overwhelming emotions in front of the patients I say, give me a second, and I just close my eyes and take a few deep breaths. Then the patients join me. And we reconnect’*.

Participants valued the principles of submission and acknowledged that they are not the *ultimate authority* or in control of every outcome, and this acceptance allows for a more compassionate, patient‐centred approach. **Emma** recognised that sometimes the need to ‘fix’ a patient got in the way of integrating spirituality into care: ‘*As nurse practitioners we’re trying to develop strategies, we’re so quick to fix things instead of making strategies with the client’.*

Whilst **Debbie** noted that for her spirituality in practice meant: ‘*To be teachable, being willing to admit my mistakes, and being receptive to the constructive criticism of others. To be open, honest, and transparent working within personal and professional limitations’.*

**Ben** echoed this saying: ‘*We have to be flexible. You have to adapt your knowledge and use it according to the needs of different people, whether they’re elderly … children, you know… we have to adapt’.*

Several participants noted that cultural humility is an essential ability to integrate spirituality into practice which include the willingness to accept and adapt to each patient’s needs to foster a relational dynamic where both practitioner and patient engage in a co‐constructed process of healing grounded in trust and mutual respect.

**Anabel** identified that, for her, spiritual care means: ‘…*to be vulnerable and authentic in the approach to those in our care,[meaning] I have to respect the code of practice for nursing professionals… the qualities we have to respect are to respect myself …respect my colleagues… respect the person who is seeking care from us, to be confidential… provide empathetic care …in line with … professional ethics and the code of conduct’.*

**Fran** recognised the need to: ‘…*train my spirit to…do the best for people and I think you can only do that by finding a way to connect with them …your immediate [task] is to make them like and respect… you enough to listen and keep coming back. That is the fundamental job. So, I guess in a way I have to appeal somehow to their spirit… that they leave and their spirits are somehow buoyed by thinking… ‘This person respected me. This person will go the extra mile for me. This person didn’t judge me… This person has a plan for me’.*

### 3.5. Theme 3: Challenges in Operationalisation of Spiritual Care

This theme focuses on the difficulties or barriers when *implementing spiritual care* frameworks in clinical settings. Participants noted that while the framework and definitions provide a foundation for spiritual care, they are often seen as difficult to operationalise. *Bureaucratic, management, legal, structural, cultural, and religious challenges* act as barriers to integrating spiritual care in daily practice.

They had difficulty assessing and planning spiritual care practices. The complexities of some frameworks make it challenging to implement in daily practice. Some noted that although some frameworks were easier to understand than others, there was not enough guidance for their application. Managers and supervisors often do not understand the importance of spiritual care, and organisations often do not support or believe in the incorporation of SCP. They asked for practical tools like A&V and measures to translate the framework into actions and best practices for holistic patient care.

**Gil** from New Zealand reflected on the frameworks: ‘*Yes, superficially [they] look good, you know. And …and superficially we could all probably buy into that. They are probably a useful guide, but leave too many unanswered questions if there to become a legal framework …’*

Additionally, participants highlighted the lack of *training for spiritual care during formal APN education*. **Charlie** said: ‘*I think it’s not taught enough, quite frankly, in nursing school they touch upon it, they look at it, but they don’, really… I don’t think they quite emphasised enough to me how important it is to foster that kind of sense of spirituality in day-to-day life, in work life …in client care’.* **Irene** from the United States of America added that to improve spiritual care there needs to be an increased awareness by APNs of its importance in holistic care.

This gap leaves many APNs finding their own pathways to address the spiritual needs of patients. One participant noted that a major challenge lies in how to teach the intangible aspects of spiritual care, such as connection and vulnerability, which are difficult to formalise into structured learning objectives. **Fran** noted: ‘[The A&V framework and SCP definition] *would just need a little bit of training… and just skilling up, I feel like this is a framework that we kind of have in the background. It’s just in our DNA, in the finish… it’s just the thing we know to do…*.*, but it’s just…. it just needs to be a thing that is so embedded in us…’*

APNs also reflected on the political, sociocultural and religious barriers they face, particularly in environments where the separation of church and state limits religious language or practices in clinical care. **Laura** reflected on working in the United States and that for her bringing in spirituality with patients is related to: ‘*Depending on their spirituality, their openness to it, the ability that I can have as a primary care provider in the United States in a federally funded facility …to help people recall the … energy of the natural earth to heal and to help …help orient them … in that healing energy that’s available to them. I have unique constraints in that …you know …I’m prohibited by the federal government from bringing in … any of my own spirituality into … my practice’*. This creates tension between the desire to provide spiritually competent care and the regulatory frameworks within which healthcare operates.

Finally, participants noted a clear need for more mentorship from supervisors, support from managers to enable integration of spirituality clinically and practical tools, such as specific exercises, curricula and assessment tools, to help them translate these frameworks into tangible actions. **Ben** noted that for APNs to embed spiritual care into practice they: ‘*Will need a sort of a spiritual supervisor…not a clinical supervisor, you need to be given some time just to switch off and calm down and, you know, reflect. Reflection needs time, the right place, the right environment and the right people’.*

### 3.6. Theme 4: Mental Health Capabilities Enabling Spiritual Care

This theme captures how spiritual care is often embedded within advanced mental health practices rather than a discrete domain of care. It explores the overlap between spiritual health and the interpersonal, emotional attributes and clinical capabilities required for effective mental healthcare. It underscores the notion that many of the core competencies for spiritual care as noted in Theme 2, are the same skills and attributes used by mental healthcare practitioners. Participants recognised that spiritual care is not a separate entity but is deeply interconnected with mental health interventions, as both require building meaningful, therapeutic relationships to address the emotional and psychological needs of the patient. Skills such as advocacy, emotional attunement and providing hope are equally important in both contexts and are essential for fostering a positive therapeutic environment.

**Maria** from Kenya reflected on how being attuned to her own spirituality helps her own mental healthcare and how for some the term spirituality is not acknowledged as such, she says: ‘*Well, basically my spiritual needs may be components that should empower or should make me mentally stable…This term spirituality is usually hidden in mental health…whereby we call it psychosocial support …you know …the term spirituality looks more religious, but if you say it’s psychosocial or psychological support, it’s ……it reflects what spirituality basically is talking about’.*

**Charlie** felt that spirituality ‘*definitely helps a lot in mental health too… and the mental health counselling and psychotherapy or things like that…we’re calling it mental hygiene’*.

This overlap suggests that APNs who integrate spirituality into practice may already possess many of the essential core skills, competencies, and attributes required for mental health, particularly in building rapport, providing psychosocial support and creating an emotionally safe space for patients.

However, the lack of explicit recognition of these practices as spiritual care may be why they were inconsistently acknowledged by all participants.

## 4. Discussion

This study explored the perceptions of APNs from 10 countries regarding their own spirituality, practices for spiritual care and their perspectives on Rogers’ [5] *Availability and Vulnerability Framework* (Figure 1) and Wattis et al.’s [9] definition of *Spiritually Competent Practice* (Figure 2). Four key themes emerged, indicating a shared understanding of spirituality as a fundamental human experience and highlighting how APNs integrate spiritual principles and attributes into patient care. Findings revealed a lack of support from managers to enable integration of spirituality, not enough mentorship from supervisors regarding spiritual care and obstacles in translating spiritual concepts into clinical practice. Mental health was interpreted by some participants as an expression of spiritual health, highlighting both the interconnection between these domains and the potential conflation arising from limited spiritual language in practice. This suggests a deep interconnection between psychological and spiritual well‐being. The themes derived from the data collectively address the research question by highlighting the complex interplay between APNs’ spiritual beliefs, their clinical practice and their engagement, or lack of engagement, with established theoretical frameworks.

Participants consistently identified a foundation of universal spiritual principles, emphasising concepts such as interconnectedness, finding meaning and transcendence. This aligns with recent research that underscores the universality of human spirituality across diverse cultural contexts and disciplines [12]. For example, APNs from various backgrounds recognised a shared sense of human dignity and the inherent value of each individual, reflecting a common spiritual ground. Other studies also emphasise the importance of recognising a patient’s holistic needs, including their spiritual dimension [13, 14]. Our findings also support the idea of spirituality as a universal human experience and not a primarily religious concept [15, 16].

APNs reported incorporating these principles into clinical practice through active listening, presence and fostering a sense of hope and meaning. This is consistent with previous findings highlighting the therapeutic value of frequent APN spiritual care assessments and interventions [17]. For instance, our participants described creating a safe space for patients to express their spiritual concerns, which aligns with the concept of ‘*being with’* the patient, a core component of spiritual care [12]. Thus, our study suggests that APNs are actively translating spiritual concepts into tangible clinical practices by supporting patients in finding meaning and hope in the context of illness, often through building trusting relationships and addressing distress beyond purely psychological symptoms. This supports the notion that spiritual care is an integral aspect of comprehensive holistic healthcare and not simply a theoretical concept.

Our findings showed that APNs were challenged in implementing and embedding spiritual care within clinical systems. This reflects the literature acknowledging difficulties in measuring, documenting and integrating spiritual care into patient records and multidisciplinary workflows [17, 18]. Participants highlighted barriers including time constraints, lack of appropriate assessment tools and limited organisational support related to staffing, space and clinical priorities. These challenges illustrate the complexity of translating spiritual care into structured, measurable and sustainable practices within healthcare systems. As in previous studies identifying systemic obstacles [17, 19, 20], participants noted the difficulty of integrating spiritual assessments into busy workflows. This underscores the need for greater managerial and institutional support to enable the effective implementation of spiritually competent practice and enhanced quality of care.

Participants also highlighted the need for standardised training and protocols to overcome the logistical and knowledge gaps. Van Leeuwen et al. [21] found that spiritual care is teachable and measurable and developed a four‐competency *EPICC Spiritual Care Education Standard*, outlining knowledge, skills and attitudes for spiritual care in nursing and midwifery students. This standard comprises four spiritual care competencies: intrapersonal spirituality, interpersonal spirituality, spiritual care: assessment and planning and spiritual care: intervention and evaluation, reflecting many elements of A&V and SCP [2].

Following the establishment of the *EPICC Spiritual Care Education Standard* an educational toolkit was developed providing resources, exercises for development and a pathway to develop personal spiritual care competencies which APNs could utilise [22]. Spiritual care competencies can be developed as part of the educational journey between clinical and personal, including self, patient and community through exercises and dialogues to enhance the journey [23]. Wattis et al. [9] advise that spiritual care competencies include personal attributes APNs use daily in caring, including being compassionate and knowledgeable, being able to grow in relationships with their patients, being open and flexible and, most importantly, being a good listener and honest [24].

Spiritual care competencies are embedded within a compassionate relationship and grounded in a person‐centred, reflective approach characterised by openness, presence and trust—principles that are fundamental to nursing. This study highlights these competencies as reflected in the participants’ experiences and perceptions. The affirmation of the A&V framework and the definition of *Spiritually Competent Practice* by APNs demonstrate that the practical application of spiritual care, through the skills and attributes outlined in the framework, enriches the understanding and implementation of spiritual care in clinical practice.

Although the APNs in this study may not have received formal education or training in providing spiritual care, they demonstrated a personal commitment to being available to their patients. They recognised how their own values created a vulnerable space in which patients felt empowered to engage in a deeper, interpersonal dimension of care—one that may not have been previously accessible to them. APNs were able to assess and identify the spiritual needs of the patient, develop a care plan and evaluate its effectiveness in promoting spiritual well‐being. These competencies are intrinsically linked to assessing, diagnosing, treating and evaluating spiritual health as a manifestation of mental health and play a pivotal role in holistic patient care. EPICC’s intrapersonal and interpersonal competencies align with the A&V framework, representing essential attributes that APNs must cultivate to effectively provide spiritual care. This type of standardised framework provides a simpler understanding and tools to create a strong foundation for developing and training spiritual competence in APNs.

While participants described a strong connection between mental and spiritual health, this finding also warrants critical examination. The tendency to interpret spiritual care through the lens of mental health may reflect not only genuine interconnection but also a lack of shared professional language and spiritual literacy within nursing practice. In the absence of clear conceptual frameworks and vocabulary, relational and psychosocial aspects of care—such as presence, empathy and meaning‐making—may be included in good mental health or general ‘good care’, rather than recognised as distinct expressions of spirituality. This conceptual blurring has been noted in the literature and risks obscuring the unique contribution of spiritual care, including its focus on transcendence, existential meaning and connection beyond the self [9, 15, 23]. The findings of this study, therefore, highlight an ongoing challenge for the profession: despite a substantial body of research, spirituality remains underdeveloped as a clearly articulated domain of practice. Strengthening spiritual language, education and conceptual clarity is essential to support APNs in distinguishing spiritual care from, while still integrating it within, mental health and truly holistic care.

Our participants perceived a strong link between mental and spiritual health, suggesting that psychological distress can manifest as spiritual unease and vice versa. This aligns with literature that recognises the interconnectedness of these dimensions in overall well‐being [25–28]. However, rather than being synonymous, spirituality and mental health represent related but distinct domains of experience. APNs described how addressing patients’ spiritual needs often led to improvements in their mental health, indicating that spiritual care can be a vital component of mental health interventions. This finding supports the growing recognition of the importance of integrating spiritual care into mental health practice, a field that has traditionally neglected this aspect of care [29–31].

## 5. Implications for Practice

The findings of this study have significant implications for APN management and practice. Firstly, educational programmes should incorporate comprehensive training and tools on spiritual competencies, assessment and interventions, equipping APNs with the necessary skills to provide holistic care [17]. Secondly, this research underscores the critical role nursing management may play in implementing effective strategies to improve the integration of APN spiritual care models and competencies. Nurse managers and healthcare institutions should develop policies and protocols that support the integration of spiritual care into routine clinical practice, including allocating time and resources for these interventions. Thirdly, APNs should recognise the potential role of spiritual care in addressing psychological distress, fostering a collaborative approach between mental health and spiritual care providers. These implications underscore the necessity for systemic changes to ensure that spiritual care is acknowledged as a vital component of patient‐centred care.

### 5.1. Limitations

This study has several limitations. Despite the larger study, the sample size was relatively small, meaning the findings are more exploratory and illustrative. While the data were deep and rich in content, we were unable to extract cultural data from all countries and cultures or make comparisons between countries. Some of the data were collected by undergraduate student research assistants supervised by a research team member, which may have impacted the richness of the data collected. Self‐selection bias may have contributed to the emphasis our participants placed on the importance of spiritual care. The reliance on self‐reported data may also introduce bias, as participants may have provided responses that seem desirable to the research team.

### 5.2. Future Research

Future research could explore the development of spiritual competence amongst newly qualified APNs and the impact of spirituality training programmes on their practice. Studies exploring patient experiences with APN spiritual care may offer valuable feedback, providing a more holistic understanding of the impact of APN spiritual care. Additional research is necessary to develop culturally sensitive spiritual assessment tools and interventions that can be effectively integrated into diverse healthcare settings and documented in patients. Comparative studies across different healthcare professions could also provide valuable insights into the interprofessional aspects of spiritual care. Finally, operational efficiency within nursing management may be studied to validate and refine evidence‐based management practices.

## 6. Conclusion

This study provides valuable insights into international APN perceptions of their own spirituality and spiritual care practices. The findings underscore the importance of integrating spiritual care into holistic patient care, highlighting the need for enhanced education, institutional support and interprofessional collaboration. By addressing the identified challenges and pursuing the suggested avenues for future research, APNs can further advance the provision of spiritually competent and patient‐centred care in their practice settings.

## Author Contributions

Abigail Kra‐Friedman: methodology, literature review, data analysis, and writing–review and editing. Nasreen Lalani: methodology, data analysis, and writing–review and editing. Gulnar Ali: methodology, supervision, data collection, and writing–review and editing. Caryn Scheinberg‐Andrews: methodology, literature review, data analysis, and writing–review and editing. Joanne Pike: methodology, literature review, data collection, and data analysis. Kimberley Lamarche: methodology and writing–review and editing. Elisabeth Gulliksen: methodology and writing–review and editing. Melanie Rogers: conceptualisation, methodology, data analysis, supervision, and writing–review and editing.

## Funding

This study received IMPACT CASE STUDY grant award from ^3^UEL, 2023, £6000—GRANT ref: A100114 (version in file).

Research was supported by University of East London ∗ A100114 (version in system).

## Conflicts of Interest

The authors declare no conflicts of interest.

## Data Availability

The data that support the findings of this study are available on request from the corresponding author. The data are not publicly available due to privacy or ethical restrictions.
